# Regulation of Synthesis and Roles of Hyaluronan in Peritoneal Dialysis

**DOI:** 10.1155/2015/427038

**Published:** 2015-10-13

**Authors:** Timothy Bowen, Soma Meran, Aled P. Williams, Lucy J. Newbury, Matthias Sauter, Thomas Sitter

**Affiliations:** ^1^Wales Kidney Research Unit, School of Medicine, College of Biomedical and Life Sciences, Cardiff University, Heath Park, Cardiff CF14 4XN, UK; ^2^Medizinische Klinik und Poliklinik IV, Klinikum der Universität München, Ziemssenstrasse 1, 80336 München, Germany; ^3^Klinikum Kempten, Robert-Weixler-Strasse 50, 87439 Kempten, Germany

## Abstract

Hyaluronan (HA) is a ubiquitous extracellular matrix glycosaminoglycan composed of repeated disaccharide units of alternating D-glucuronic acid and D-N-acetylglucosamine residues linked via alternating *β*-1,4 and *β*-1,3 glycosidic bonds. HA is synthesized in humans by HA synthase (HAS) enzymes 1, 2, and 3, which are encoded by the corresponding *HAS* genes. Previous in vitro studies have shown characteristic changes in HAS expression and increased HA synthesis in response to wounding and proinflammatory cytokines in human peritoneal mesothelial cells. In addition, in vivo models and human peritoneal biopsy samples have provided evidence of changes in HA metabolism in the fibrosis that at present accompanies peritoneal dialysis treatment. This review discusses these published observations and how they might contribute to improvement in peritoneal dialysis.

## 1. Hyaluronan: Multiple Functions and Clinical Significance

Hyaluronan (HA) is a linear glycosaminoglycan associated most commonly with the extracellular matrix (ECM). HA was first isolated from the optic vitreous [1] and is composed of tandem repeats of a D-glucuronic acid and D-N-acetylglucosamine disaccharide motif linked via alternating *β*-1,4 and *β*-1,3 glycosidic bonds [2]. Unique amongst glycosaminoglycans, HA is unsulfated and contains no epimerised uronic acid residues. To export HA to the ECM, the HA synthase (HAS) proteins traverse the plasma membrane and act as glycosyltransferases, combining precursor UDP-glucuronic acid and UDP-N-acetylglucosamine to form HA. HA polymers are thus synthesised at the HAS active site on the intracellular side of the membrane and exported instantaneously as linear, unaltered polymers [3].

HA was thought initially to be an inert, space-filling molecule [4]. More recent analyses, however, have shown that HA is a multifunctional molecule for which a number of key roles have already been identified during and following development. These inter- and intracellular functions include roles in cell migration, tumour invasion, and cellular response to injury (e.g., [5–10]).

The importance of HA in the ECM is underlined by the expanding range of pathological contexts in which modified or aberrant HA metabolism appears to play a role. These include autoimmune renal injury, fibrosis of the kidney and other large organs, diabetic nephropathy, malignancy, osteoarthritis, and pulmonary and vascular disorders, along with other immune and inflammatory diseases (e.g., [6, 9–30]). HA deposition is characteristic of peritoneal fibrosis subsequent to dialysis treatment [31, 32]. HA has also been implicated in regenerative processes such as wound healing in the peritoneum and elsewhere [33–39] and as a key immune mediator [20, 21]. Upregulation of HA synthesis has also been reported in inflammation that occurs commonly as a consequence of treatment of renal failure by peritoneal dialysis (PD) [32, 33]. The focus of this review will be on the regulation and function of HA in PD.

## 2. Regulation of HA Synthase (HAS) Expression

HA is synthesised by the enzymes HAS1-3. These proteins are encoded by the corresponding* HAS* genes* HAS1*,* HAS2*, and* HAS3*, with each human gene located at a discrete autosomal locus [40].

The human peritoneal mesothelial cells (HPMCs) that line the peritoneal membrane synthesise HA as a normal constituent of peritoneal effluent, and this synthesis is upregulated during periods of peritonitis [41]. In an in vitro model of peritoneal wound healing [42], mechanical disruption of HPMC monolayers led to upregulated HAS2 transcription together with an increase in HA synthesis [33].

However, despite the above array of pathological and physiological functions already ascribed to HA, comparatively little is known about the regulation of human HAS expression in peritoneal inflammation and fibrosis.

We began our studies on the regulation of HAS expression by defining genomic structures for each human* HAS* gene [43]. As part of this study, we prepared luciferase reporter constructs spanning approximately 0.5 kb of genomic DNA upstream of each putative HAS transcription start site (TSS) [43, 44]. Each sequence showed significant promoter ability to drive transcription of the luciferase gene [43].

To locate the HAS2 promoter, we carried out HAS2-specific 5′-rapid amplification of cDNA ends (5′RACE) on polyadenylated RNA extracted from renal proximal tubular epithelial cells and located the TSS 0.130 kb upstream of the 5′ end of HAS2 reference mRNA sequence NM_005328 [45]. We then generated luciferase reporter vectors bearing nested fragments spanning the first 0.8 kb upstream of this new TSS [45, 46]. Luciferase analysis showed consistent promoter activity mediated by a minimum-sized fragment of 0.121 kb, within which we identified promoter sequences conserved in selected mammals [45, 46]. Similar methods have recently been used to identify the human HAS3 promoter [47].

Using electrophoretic mobility shift and supershift data, we then demonstrated binding of transcription factors Sp1 and Sp3 to three sites immediately upstream of the HAS2 TSS [48]. Luciferase analysis of mutated reporter constructs was abrogated, while RT-qPCR analysis following siRNA knockdown of either transcription factor significantly reduced the level of HAS2 transcription [48]. Chromatin immunoprecipitation analysis of this locus has since been used to analyse HAS2 transcriptional induction by retinoic acid and tumour necrosis factor-*α* [49].

The tetraexonic, long noncoding RNA transcript HAS2-AS1 is transcribed from the opposite genomic DNA strand to HAS2 mRNA at 8q24.13 [50]. The second exon of HAS2-AS1 shares partial sequence complimentary with HAS2 exon 1, and HAS2-AS1 can therefore be described as a “natural antisense” to HAS2 [50]. In osteosarcoma cells, transcription of HAS2 mRNA synthesis and subsequent HA production are downregulated by HAS2-AS1 [50]. By contrast, in renal proximal tubular epithelial cells, we showed that HAS2-AS1 expression augments and/or stabilises HAS2 mRNA and detected cytoplasmic HAS2:HAS2-AS1 RNA duplexes [51]. In aortic smooth muscle cells, HAS2-AS1 also upregulates HAS2 expression and mediates posttranscriptional modification of HAS2 by O-GlcNAcylation [52].

We have also identified the HAS1 TSS, adding a further 26 nucleotides to reference sequence NM_001523, and analysed the upstream HAS1 promoter region in renal proximal tubular epithelial cells [46, 53], but a full characterisation of factors regulating HAS expression in HPMCs has not been carried out. In addition, little is known about HPMC expression of long noncoding RNAs (including HAS2-AS1) and of microRNAs, both of which are highly likely to regulate HAS expression. Indeed, understanding the transcriptional and posttranscriptional mechanisms regulating HPMC HAS expression will provide useful information on the control of HA synthesis during PD and has the potential to inform future approaches to antifibrotic PD therapy.

## 3. Synthesis of HA by Peritoneal Mesothelial Cells

HA is an important component of the HPMC ECM and is also produced by fibroblasts and macrophages in the peritoneal cavity [54–56]. According to in vivo findings, HA levels are increased in peritoneal dialysate during peritonitis [54]. It has also been shown in vitro that the synthesis of HA in mesothelial cells is enhanced by various inflammatory mediators including prostaglandin E2, PDGF, transforming growth factor-beta1, tumour necrosis factor-alpha (TNF-*α*), and interleukin-6 (IL-6), with IL-1*β* producing the strongest effect [41, 55, 57].

HA is found predominantly in connective tissue where the polymer chain is bound to interacting molecules such as cell surface receptor CD44, the receptor for HA-mediated motility, and proteoglycans including aggrecan and versican [58, 59].

Under homeostasis, HA polymers are typically between 2,000 and 25,000 disaccharide units in length, and these chains have been sized at 2–25 *μ*m [11]. Yung and Chan [32] have ably summarised much previous work on the properties and effects of low and high molecular weight HA. Despite this sizeable body of accumulated data, attribution of functions dependent on the number of disaccharide repeat units in the HA polymer remains controversial. Understanding the potential functional differences is complicated by the fact that HA can be digested by the hyaluronidase (HYAL) enzymes encoded by the* HYAL* multigene family [60] and by HA degradation at different sites. An early in vivo study in which radiolabelled HA was injected into rabbit knee joints showed degradation locally and in the liver [61].

The biological effects of adding exogenous HA preparations to cultured HPMCs have been studied in vitro in numerous cell culture systems. Yung and colleagues [33] showed that addition of HA accelerated in vitro healing of wounded HPMC monolayers in a dose-dependent manner between 50 and 3300 ng/mL. Mediation of these proliferative HA effects by interaction with CD44 remains unproven [62]. The key role played by HA in the process of remesothelialisation was confirmed in a further in vitro model of HPMC cell migration [63].

When HPMCs were exposed to spent dialysates supplemented with 0.1 or 0.5 mg/mL high molecular weight HA, synthesis of chemokine monocyte chemoattractant protein (MCP-1), adhesion molecule soluble intercellular adhesion molecule (s-ICAM), vascular endothelial cell growth factor (VEGF), and fibronectin was significantly reduced [64]. However, these differences were not observed in vivo when the same inflammatory mediators were measured in drained dialysate patient samples [64]. In accordance with the above in vitro findings, high molecular weight HA also inhibits the nuclear factor-kappa B- (NF-*κ*B-) dependent synthesis of cytokines IL-1*α*, IL-6, and TNF-*α* in mouse macrophage line J774 [65].

Since HA alters the fibrinolytic properties of numerous cell types [66], we investigated the effect of HA on the synthesis of tissue plasminogen activator (tPA) and plasminogen activator-1 (PAI-1) in HPMCs. Only very high concentrations of HA (>50 mg/dL) downregulated fibrinolytic HPMC activity by decreasing t-PA synthesis, but changes in t-PA and PAI-1 expression were not observed at concentrations up to 30 mg/dL [66]. A subsequent study has shown that monocyte/macrophage system cells interfered with HA-associated changes in the fibrinolytic capacity of HPMCs treated with lipopolysaccharide [67].

HA fragments activate nitric-oxide synthase in murine macrophages through an NF-*κ*B-dependent mechanism, increasing expression of chemokines macrophage inflammatory protein-alpha (MIP-1*α*), MIP-1*β*, and MCP-1 [68, 69]. In renal cortical tubular cells the synthesis of adhesion molecules ICAM-1 and vascular cell adhesion protein-1 (VCAM-1), and MCP-1, were upregulated following stimulation with low molecular weight HA [70]. Similarly, we found that HA fragments of approximate molecular mass 1–7 × 10^5^ Da induced the synthesis of the chemokines MCP-1 and IL-8 in HPMCs [71]. The upregulation of these chemokines was preceded by an increase in NF-*κ*B and activating protein-1 DNA binding activity in HPMCs [71].

Breborowicz and coworkers found changes in HPMC synthetic activity following exposure to dialysis fluids, including downregulated HA synthesis [72]. Glutathione precursor L-2-oxothiazolidine-4-carboxylic acid prevented this effect, suggesting that it may be driven by glucose-induced free radicals during PD [72]. Chronic exposure of HPMCs to glucose or N-acetylglucosamine showed the latter to be more biocompatible, despite the fact that it upregulated HA synthesis [73]. Treatment with peroxisome-proliferator activator receptor-gamma agonist ciglitazone decreased endometrial cell attachment to HPMC line LP9 and decreased LP9 attachment to HA-treated tissue culture plate wells [74]. HPMC senescence in vitro was accelerated by glucose but not N-acetylglucosamine [75].

In summary, the effects of in vitro HA application to HPMCs are complex and dependent on HA molecular weight. High molecular weight HA appears to have anti-inflammatory and preservative effects, while low molecular weight HA stimulates proinflammatory processes. However, as several studies have failed to discriminate clearly between the effects of HA of different sizes, an unambiguous interpretation of these data is challenging, and new data will be required to resolve this issue.

## 4. HA in Peritoneal and Endothelial Glycocalyces

Emerging evidence suggests that HA at the mesothelial cell surface contributes significantly to the peritoneal glycocalyx [32, 76–78]. This structure performs a number of roles including protection and lubrication, and denudation during PD and/or injury is likely to accelerate HPMC and peritoneal damage and thereby treatment failure [32, 77]. HA is also a key component of the endothelial glycocalyx, contributing significantly to its permeability [79, 80], and a recent study has investigated the potential importance of the systemic microvascular endothelial glycocalyx as a transport barrier during PD [81].

## 5. Intraperitoneal In Vivo Administration of HA to Prevent Surgery-Induced Adhesions

Postoperative adhesions are a frequent outcome of abdominal surgery and may lead to bowel obstruction, chronic pelvic pain, infertility, and technical difficulties in further surgical procedures [82]. Both in humans and in animal models, intraperitoneal (IP) administration of HA has been tested in an attempt to prevent the formation of postsurgical adhesion. Numerous HA preparations for IP application are commercially available or in development. Interpretation of the diverse outcomes following their use is not straightforward, as is clear from the studies described below.

A number of prospective randomized trials have been carried out to determine the efficacy of HA-based adhesion-preventing agents. One such study analysed the antiadhesion efficacy of a 0.5% ferric hyaluronate gel in severe peritoneal trauma caused by bipolar coagulation in a laparoscopic rat model. Adhesion scores were decreased significantly, but none of the animals was free of adhesions, and the authors did not show a significant difference between the HA gel treatment and the use of the adhesion-preventing agents Ringer's lactate solution and 4% icodextrin solution [83]. In a prospective randomized study of peritoneal laparoscopic resection in rabbits using 0.5% ferric hyaluronate gel, saline, or control, no differences in adhesion scores and number of animals with adhesions were reported [84]. By contrast, a prospective randomized multicentre study in humans showed that a glycerol-HA/carboxymethylcellulose membrane effectively reduced intra-abdominal adhesions in patients who underwent proctocolectomy and ileal-pouch-anal anastomosis [85]. However, the increase in infectious complications caused the manufacturer not to market this product [85].

Considerable research efforts have been devoted to analysis of membranes composed of HA and carboxymethylcellulose (HA/CMC; commercially available as Seprafilm) both alone and in conjunction with additional agents, while other treatments have also been tested. For instance, Nilsson and colleagues [86] investigated the use of HAPXL01, a novel polypeptide derived from human lactoferrin, in the sidewall-defect cecal abrasion model in the rat [86]. An HA-based formulation of HAPXL01 inhibited scar formation, prohibiting inflammation and promoting fibrinolysis and significantly reducing adhesion formation without affecting wound healing [86].

In the first of two studies by Lim and coworkers, HA/CMC Seprafilm mediated effective reduction in adhesion formation by peritoneal ischemic buttons created either side of a midline incision that was limited to the site of application [87]. Irrespective of the bioresorbable material at predicted adhesion sites, peritoneal adhesions formed readily at unprotected sites [87]. In a later study, coadministration of neurokinin 1 receptor antagonist significantly augmented the effect of the HA/CMC membrane on adhesion prevention from experimentally induced peritoneal ischaemic buttons, and combined use of these treatments reduced adhesion formation both at the site of application and at distal sites [88].

In a rat laparotomy/cecal injury model, IP-administered atorvastatin proved to be equally as effective as Seprafilm in the prevention of postoperative adhesion, but there was no additive effect when both treatments were combined [89].

Data on HA-based membranes has not always been positive, however. The study of Economidou and coworkers [90] set out to evaluate the effects of administration of two (unidentified) commercial membranes: a thicker membrane composed of macromolecular polysaccharides and a thinner HA-hydroacid methylcellulose-based membrane. The use of the former resulted in elevated serum creatinine and urea levels, tubular epithelial cell vacuolization, and mild interstitial infiltration [90]. These lesions were milder when the HA-based membrane was used, and serum creatinine did not change [90].

Use of HA/CMC powder and film applied either directly or contralaterally was compared in a rat peritoneal sidewall defect model and a rabbit cecal abrasion/sidewall defect model [91]. Both additives reduced adhesions to the same degree on direct application, while powder alone was effective on remote application but did not inhibit wound healing [91]. In a severe adhesion model in which 1 cm^2^ of intra-abdominal wall was excised and* n*-butyl-2-cyanoacrylate was applied, Hwang and colleagues [92] compared HA/sodium CMC gel (Guardix-sol), 4% icodextrin, and Seprafilm. Scoring for both fibrosis and adhesion showed a significant reduction in both when Seprafilm alone was used [92].

A comparison of IP-administered linezolid with Seprafilm [93] found both to be significant in reducing formation of rat peritoneal adhesions following sterile antimesenteric (side) surface cecal abrasion, when compared to controls. Similarly, both IP lovastatin and Seprafilm were equally effective in preventing postoperative intra-abdominal adhesions of cecal, ileal, and uteral abrasions [94]. Observations from a rabbit model showed that the combined use of 3% trehalose solution and Seprafilm had additive effects in the prevention of adhesion formation [95]. A recent study using a biodegradable HA-based hydrogel formed in situ showed a promising and significant reduction in adhesion formation [96].

In summary, the use of a variety of HA and HA-derived preparations used in past studies complicates the process of data interpretation. However, a significant body of evidence now supports the use of HA/CMC agents, and the utility of intraperitoneally administered HA has potential for future development.

## 6. Animal Studies on PD Examining In Vivo HA Application

Where appropriate, selected data from the studies discussed below are summarized in Table 1. The therapeutic application of the addition of HA to PD fluid is based on the assumption that HA is lost from the peritoneal cavity during PD [97]. In PD patients, IP production of HA increases during episodes of peritonitis [41, 98], and with the duration of PD therapy the HA concentration rises in the effluent of PD patients [99]. Consequently, the first animal studies were performed to determine the role of HA in peritoneal function during PD [97]. A number of studies using IP-soluble HA administration have been described, the majority being in animal models.

### 6.1. Effects of HA-Containing Fluid on PD Transportation Characteristics

Significantly lower transperitoneal protein equilibration for albumin and for total protein in rats receiving a Dianeal solution containing 10 mg/dL HA twice daily for 4 weeks has been reported [97], and these data have been supported by the results of other studies [100, 101]. Furthermore, the total drained volume after a 4-hour dwell was significantly higher in the HA group, yielding a positive net ultrafiltration (UF) in the HA group versus a negative net UF in the control group [97].

The above effect was based mainly on a decreased peritoneal fluid absorption rate, which was demonstrated independently following 4-hour HA solution dwell, together with increased urea clearance [102]. The same authors showed that there was no difference in the transcapillary UF rate (Qu) between different concentrations and various molecular weights of HA in control groups or even a reduced Qu in one HA group receiving 4 MD molecular weight HA [103]. They also reported that the effect of HA on peritoneal fluid absorption and net UF appeared to be both size- and concentration-dependent [104] and noted that this HA-mediated effect was potentially useful to prevent a decrease in net UF caused by increased peritoneal dialysate fill volume [105]. With respect to small solute clearance, it was shown that HA administration resulted in a significantly increased urea clearance [100, 105].

Rosengren et al. [106] provided evidence that both small solute transfer and glucose-induced osmotic water transfer (using a 3.86% glucose-based solution) was not influenced by HA supplementation of dialysis fluid. HA did, however, reduce backfiltration of fluid from peritoneum to plasma by forming a “filter-cake” [106]. These researchers also found that hyaluronidase incubation resulted in a 78% reduction of HA in the superficial layer of the peritoneal membrane which did not produce any significant changes in solute and fluid transport across the peritoneal membrane [107].

### 6.2. Effect of HA-Containing Fluids on Peritoneal Inflammation

The effect of HA on peritoneal inflammation has also been analysed in animal studies. IP administration of HA in rats reduced the percentage of neutrophils in PD effluent, but the total cell number in the effluent did not change [97]. This study also reported lower levels of TNF-*α* and MCP-1 in rats treated with HA-containing PD solution in comparison to rats treated with Dianeal [97]. In a model of lipopolysaccharide-induced peritonitis in rats, 10 mg/dL HA administration reduced loss of UF and provided a greater creatinine clearance [108]. Furthermore, the presence of HA led to increased dialysate interferon-*γ* levels, whereas elastase levels decreased [108].

### 6.3. HA Absorption and Metabolism after IP Application

Breborowicz and coworkers have investigated the absorption and metabolism of 10 mg/dL HA in Dianeal after IP administration [100]. After 8 hours, one quarter of the HA had been absorbed from the peritoneal cavity and peritoneal tissue, and plasma HA concentrations were significantly increased to 116% in peritoneal tissue and 435% in plasma [100]. HA levels returned to normal within 24 hours after IP administration in both healthy and uremic rats [100].

### 6.4. Histological Evaluation of the Peritoneal Membrane after HA Application

Only one study has provided histological evaluation of the peritoneal membrane following HA administration, showing a similar increase in the thickness of the peritoneal interstitium in rats exposed to HA and in control animals [97].

## 7. HA Supplementation in Human PD

Moberly et al. [109] examined 13 patients in a prospective randomized crossover study. The PD solutions investigated were Dianeal alone or supplemented with either 0.1 g/L HA or 0.5 g/L HA. Each 6-hour dialysis exchange was separated from the other exchanges by a 2-week washout period. The authors did not report any adverse events related to HA administration. HA application did not result in significant changes in net UF or peritoneal volume profiles, but mean net UF tended to be slightly higher during treatment with HA-containing fluid. Peritoneal fluid reabsorption also tended to be lower during the HA treatment, but the differences were not significant. Solute clearances, dialysate/plasma ratios, and mass transfer area coefficients for sodium, urea, creatinine, albumin, and glucose were similar for the three treatment solutions [109]. While these data failed to reach significance, only 10 patients completed the study. In addition, HA concentrations exceeding 0.5 g/L were not used due to increased viscosity resulting in significantly increased filling and drainage times [109]. It remains possible that a longer application period than one test solution every 2 weeks might have been more effective.

A recent review by Cho and colleagues of randomized control trials and quasirandomised control trials in adults and children compared the effects of biocompatible PD solutions [110]. These authors concluded, on the basis of what they referred to as “generally suboptimal quality evidence,” that the use of neutral pH, low glucose degradation product (GDP) solutions led to greater UF and renal preservation without statistically significant effects of peritonitis, technique failure, or patient survival [110].

### 7.1. HA as a Biomarker of Inflammation in PD

In more recent work on rat models and in PD patients, HA has been used as a biomarker to monitor PD progress.

Wieczorowska-Tobis and colleagues showed reduced HA concentration in dialysate from neutral pH and low GDP PD solutions when compared with conventional solutions in rats [111], while a study on uremic rats reported increased HA and MCP-1 in peritoneal lavage fluid [112]. A rise in the parietal peritoneal concentrations of HA and collagen followed the use of PD solutions containing N-acetylglucosamine or glucose [113]. Increased peritoneal cell influx and HA synthesis, together with increased neutrophil counts and decreased mast cell/eosinophil numbers, were observed in rats following PD, and these affects were not changed by the presence of unfractionated or low molecular weight heparin [114]. Reduced HA production was reported in a rat model using bone morphogenetic protein-7 (BMP-7) over a 5-week period, suggesting decreased fibrosis [115]. Use of icodextrin in PD solutions resulted in increased patient dialysate HA compared to glucose/lactate solution, suggesting increased subclinical inflammation [116].

Breborowicz et al. [64] examined the effect of Dianeal alone or Dianeal with HA supplementation of 0.1 or 0.5 g/L. Exchanges at 6 hours were performed with each of the fluids randomly at 2-week intervals. Patient dialysate nitrite concentration (as an index of NO production) was significantly higher after dialysis exchange performed with HA 0.5 g/L, but concentrations of MCP-1, s-ICAM 1, VEGF, and fibronectin were similar after exchanges with the HA-supplemented dialysate fluids and did not differ from Dianeal alone. When cultured HPMCs were exposed for 24 hours to these dialysates, the HA-containing fluids inhibited the synthesis of MCP-1, s-ICAM, VEGF, and fibronectin, accelerating the growth rate of proliferating cells [64].

Use of icodextrin or amino acid-based PD solutions did not result in significant changes in HA production in human dialysates [117], whereas upregulated HA synthesis was reported in whole peritoneal samples from long-term PD patients [118]. Recent reports have shown increased HA after 8 weeks of PD [119] and upregulated expression of endothelial HA receptor-1 (LYVE-1) in human dialysate effluents, peritoneal tissues, and HPMCs [120]. Most recently, Yung and colleagues analysed data from a comprehensive panel of fibrotic and inflammatory biomarkers, including HA, in a randomised prospective study of 80 PD patients [121]. This study compared a low-glucose treatment protocol of Physioneal, Extraneal, and Nutrineal with a control group treated with Dianeal; the biomarker data suggested better preservation of membrane integrity in the multitreatment group [121].

## 8. Summary

The roles of HA in peritoneal biology, fibrosis, and dialysis require further investigation. A more complete understanding of the regulation of peritoneal HAS expression and HA synthesis, and of peritoneal and mesothelial responses to exogenous HA, has the potential to provide new tools with which PD treatment and prevention of surgical adhesions are improved. The manipulation of HAS expression and HA metabolism via long noncoding RNAs such as HAS2-AS1 and/or microRNAs, together with recent advances in molecular analysis techniques, hold much promise in these contexts [50–52, 122–128].

## Figures and Tables

**(a):** 

Study	Organism	HA concentration and application	HA Effects on UF	Other HA Effects
Wang et al., 1997 [102]	Rat	Single 4 h dwell with HA 0.005% and 0.01%	Increased UF, mainly through decreased peritoneal fluid absorption	Increased peritoneal clearance of urea

Wang et al., 1999 [103]	Rat	Single 4 h dwell with HA 0.01%	Increased net UF, mainly by decreased peritoneal fluid absorption	Increased peritoneal clearance of urea. HA may prevent decreased net UF caused by an increased dialysate fill volume

Wang et al., 1999 [104]	Rat	Single 4 h dwell with HA in various concentrations (0.01–0.5%) and molecular weights (MW; 85 kDa–4 MDa)	Increased size and concentration of HA resulted in decreased peritoneal fluid absorption. Low concentrations of high MW HA might decrease transcapillary UF rate	

Połubinska et al., 2000 [97]	Rat	High MW HA 10 mg/dL twice daily for 4 weeks	Total drained volume in HA group was significantly higher (positive net UF in HA group versus negative net UF in control group)	Clearance of total protein and albumin tended to be lower; clearance of urea and creatinine tended to be higher. Significantly decreased percentage of IP neutrophils and levels of MCP-1 and TNF-*α*

Guo et al., 2001 [101]	Rat	0.025% HA in a 4 h dwell for 1 week	Decreased peritoneal fluid absorption (similar to native animals)	Significant decrease in protein transportation rate

Breborowicz et al., 2001 [100]	Rat	One infusion of 10 mg/dL HA for a 1–8 h dwell	Net UF was significantly greater at 4, 6, and 8 h compared to controls	During 8 h exchange, creatinine clearance was significantly higher and total protein clearance significantly lower. After 8 h, 25.7% HA absorbed from peritoneal cavity and peritoneal tissue HA increased to 117%; plasma HA levels increased to 435%. Plasma HA normalized within 24 h in uremic and nonuremic animals

Breborowicz et al., 2001 [108]	Rat	Acute peritonitis induced with lipopolysaccharide; HA at 10 mg/dL; 4 and 8 h dwell	Significant reduction in loss of UF	Significantly increased creatinine clearance. Greater dialysate interferon-*γ*-levels and less pronounced elastase levels

Moberly et al., 2003 [109]	Human	Prospective randomized crossover study with 6 h application of Dianeal and Dianeal containing HA (0.1 and 0.5 g/L). 2-week washout analysed after exchange	No significant differences in net UF or peritoneal volume profiles	No adverse effects of HA

Breborowicz et al., 2004 [64]	Human	One 6 h dwell with 13.6 g/L glucose-based solution ±0.1 and 0.5 g/L of exogenous high MW HA. 2-week application intervals		Significant increased concentration of nitrites in HA 0.5 g/L supplemented dialysate. No difference in concentrations of MCP-1, s-ICAM1, EGF, and fibronectin

**(b):** 

Study	Organism	PD regime (not HA)	Other HA effects
Wieczorowska-Tobis et al., 2004 [111]	Rat	2 daily injections of 4.25% glucose-containing PDF for 6 weeks. PDFs tested: CAPD3 (single-chamber bag, low pH, and high GDP), CAPD3 pH 7.4 (single-chamber bag, neutral pH, and high GDP), CAPD3 balance (double-chamber bag, neutral pH, and low GDP)	Reduced concentrations of protein and HA in dialysate. Introduced PD fluids with physiologic pH and low GDP level producing less irritation to the peritoneal membrane, better preserving its structural integrity

Zareie et al., 2005 [112]	Rat	Uraemic and control rats received daily 10 mL conventional glucose containing PD fluid, via peritoneal catheters during a 6-week period	Increased MCP-1 and HA levels in peritoneal lavage fluid

Flessner et al., 2006 [113]	Rat	Filtered solutions with 4% N-acetylglucosamine (NAG) or 4% glucose (G) IP injected daily in 2–300 g rats compared with controls (C). At 2 months, transport studies using chamber affixed to parietal peritoneum determined small-solute and protein mass transfer, osmotic filtration, and hydraulic flow	Tissue analysis showed treatment effects on tissue HA (microg/g: C, 962 ± 73; G, 1,169 ± 69; NAG, 1,428 ± 69; and *p* < 0.05) and collagen (microg/g: C, 56.9 ± 12.0; G, 107 ± 12; NAG, 97.6 ± 11.4; and *p* < 0.05) but not sulfated glycosaminoglycan

Schilte et al., 2009 [114]	Rat	Used 10 mL PD fluid daily, ±unfractionated heparin, or low MW heparin in PD fluid (1 mg/10 mL) IP via mini access port, untreated control rats. At 5 weeks, peritoneal transport was tested; tissues and peritoneal leukocytes were sampled	Increased peritoneal cell influx and HA production (*p* < 0.02) as well as an exchange of mast cells and eosinophils for neutrophils after PD treatment observed in PD rats

Loureiro et al., 2010 [115]	Rat	Over 5 weeks, rats instilled daily using PD fluid ± BMP-7	rhBMP-7-treatment did not significantly affect any of these processes induced by PD fluid exposure, except for a tendency to reduce HA production (*p* = 0.054), suggesting decreased peritoneal fibrosis

Rosengren et al., 2013 [119]	Human	After 8 weeks PD, interstitial fluid (IF) from peritoneum was isolated via centrifugation; IF and plasma were analyzed for cytokine content and colloid osmotic pressure	IF colloid osmotic pressure decreased significantly in PD group, while collagen and HA content was increased

Kinashi et al., 2013 [120]	Human	Role of the lymphangiogenesis mediator VEGF-C analysed in human dialysate effluents, peritoneal tissues, and HPMCs	Peritoneal tissue from patients with UF failure expressed higher levels of VEGF-C, LYVE-1, and podoplanin mRNA and contained more lymphatic vessels than tissue from patients without UF failure
